# Bis[2-acetyl-3-methyl­pyrazine (2-hydroxy­benzo­yl)hydrazonato]zinc(II) monohydrate

**DOI:** 10.1107/S1600536808009707

**Published:** 2008-04-10

**Authors:** Xi-Shi Tai, Yi-Min Feng, Hua-Xiang Zhang

**Affiliations:** a Department of Chemistry, Weifang University, Weifang 261061, People’s Republic of China

## Abstract

In the title compound, [Zn(C_14_H_13_N_4_O_2_)_2_]·H_2_O, the Zn^II^ centre is six-coordinated by four N and two O donors of two 2-acetyl-3-methyl­pyrazine (2-hydroxy­benzo­yl)hydrazonate ligands, and forms a distorted octa­hedral structure.

## Related literature

For related literature, see: Herzfeld & Nagy (1999 ▶); Xi-Shi & Yi-Min (2008 ▶); Tai *et al.* (2003 ▶); Tai *et al.* (2008 ▶); Tai, Feng, Kong, Wang & Tan (2007 ▶); Tai, Yin & Feng (2007 ▶); Tai, Yin & Hao (2007 ▶); Tai, Yin, Feng & Kong (2007 ▶); Wang *et al.* (2007 ▶).
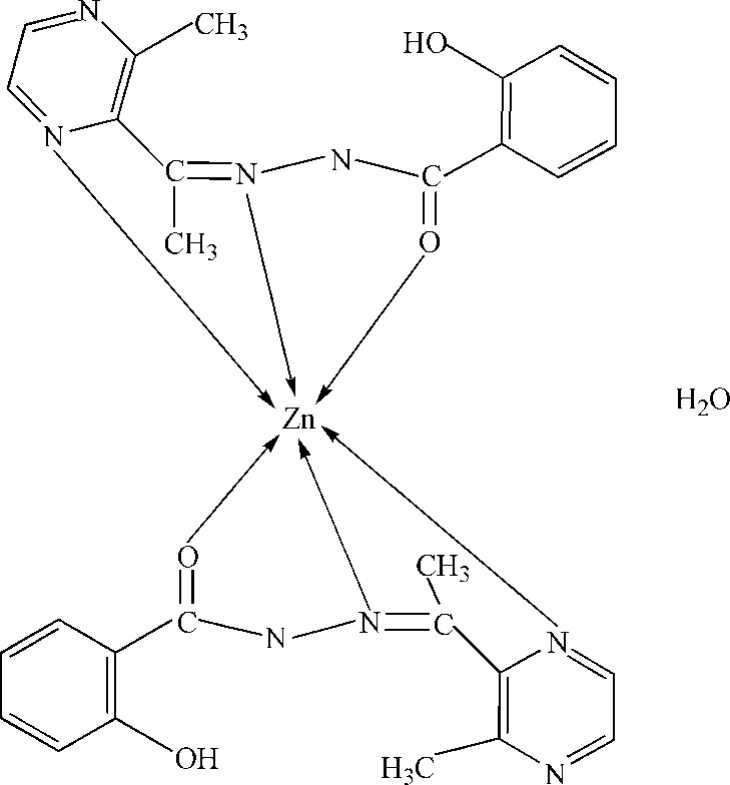


## Experimental

### 

#### Crystal data

[Zn(C_14_H_13_N_4_O_2_)_2_]·H_2_O
*M* *_r_* = 621.95
Monoclinic, 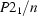

*a* = 9.3794 (10) Å
*b* = 22.814 (2) Å
*c* = 13.9407 (14) Å
β = 106.402 (2)°
*V* = 2861.6 (5) Å^3^
*Z* = 4
Mo *K*α radiation
μ = 0.91 mm^−1^
*T* = 298 (2) K
0.32 × 0.20 × 0.16 mm

#### Data collection

Bruker SMART CCD area-detector diffractometer
Absorption correction: multi-scan (*SADABS*; Bruker, 2000 ▶) *T*_min_ = 0.759, *T*_max_ = 0.868
14227 measured reflections
5043 independent reflections
2831 reflections with *I* > 2σ(*I*)
*R*_int_ = 0.076

#### Refinement

*R*[*F* ^2^ > 2σ(*F* ^2^)] = 0.047
*wR*(*F* ^2^) = 0.128
*S* = 1.01
5043 reflections
379 parameters
H-atom parameters constrained
Δρ_max_ = 0.38 e Å^−3^
Δρ_min_ = −0.35 e Å^−3^

### 

Data collection: *SMART* (Bruker, 2000 ▶); cell refinement: *SAINT* (Bruker, 2000 ▶); data reduction: *SAINT*; program(s) used to solve structure: *SHELXS97* (Sheldrick, 2008 ▶); program(s) used to refine structure: *SHELXL97* (Sheldrick, 2008 ▶); molecular graphics: *SHELXTL* (Sheldrick, 2008 ▶); software used to prepare material for publication: *SHELXTL*.

## Supplementary Material

Crystal structure: contains datablocks global, I. DOI: 10.1107/S1600536808009707/at2559sup1.cif

Structure factors: contains datablocks I. DOI: 10.1107/S1600536808009707/at2559Isup2.hkl

Additional supplementary materials:  crystallographic information; 3D view; checkCIF report

## Figures and Tables

**Fig. 1.**
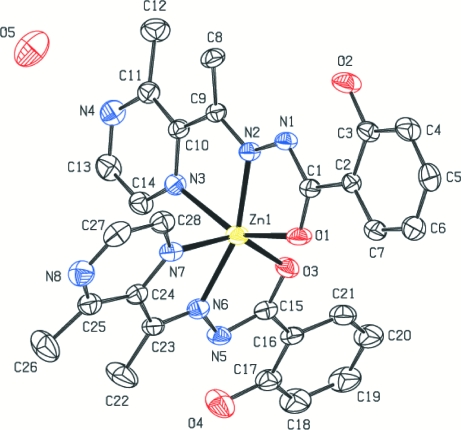
The molecule of the title compound, with 30% probabiility ellipsoids.

## supplementary crystallographic information

### Comment

Schiff-base ligands are able to coordinate metals through imine nitrogen and another group, usually linked to the aldehyde. Modern chemists still prepare Schiff-bases, and nowadays active and well designed Schiff-base ligands are considered to be "*privileged ligands*" (Tai & Feng, 2008; Tai, Feng, Kong, Wang & Tan, 2007; Tai *et al.*,  2008; Tai, Yin & Feng, 2007; Tai, Yin, Feng & Kong, 2007; Tai, Yin & Hao, 2007; Tai *et al.*,  2003; Wang *et al.*,  2007). In fact, Schiff bases are able to stabilize many different metals in various oxidation states. Schiff bases and their metal complexes play a key role in understanding the coordination chemistry of transition metal ions (Tai *et al.*,  2003). In particular, the bidentate ligands containing imine groups have been used as modulators of structural, electronic, antitumor activity and fluorescence properties of transition metal centres (Herzfeld & Nagy, 1999). In order to investigate further the coordination and the properties of zinc complexes with Schiff bases ligands, as parts of our studies on the synthesis, characterization and properties of Schiff bases ligands and their metal complexes, we herein report the synthesis and structural characterization of a new zinc complex, Zn(2-acetyl-3-methylpyrazine salicyloyl hydrazone), (I).

The title compound consists of neutral complex, Zn[2-acetyl-3-methylpyrazine (2-hydroxybenzoyl)hydrazone]. The Zn^II^ center is six-coordinate with four N and two O donors of 2-acetyl-3-methylpyrazine (2-hydroxybenzoyl)hydrazonate, and forms a distorted octahedron structure. The Zn—O and Zn—N bond lengths are in the ranges 2.055 (3)–2.079 (3) and 2.239 (4)–2.283 (3) Å, respectively. The Zn—O bond lengths are much shorter than Zn—N, which shows that the Zn—O bonds are stronger than the Zn—N bonds.

### Experimental

The title compound was prepared as following: 1 mmol of Zn^II^ acetate was added to the solution of 2-acetyl-3-methylpyrazine salicyloyl hydrazone (1 mmol) in a 10 ml of CH_3_CH_2_OH. The mixture was continuously stirred for 3 h at refluxing temperature, evaporating some ethanol, then the product was collected by filtration, yield 68%. The single-crystal suitable for X-ray determination was obtained by evaporation from ethanol solution after two weeks. A ethanol solution of the title compound was slowly evaporated and pale crystals were obtained after a weeks.

### Refinement

The positions of all H atoms were fixed geometrically [C—H = 0.93 for aromatic H and 0.96 Å for methyl H , O—H = 0.82 for hydroxy group and O—H = 0.85 Å for water molecules] and refined as riding with *U*_iso_(H) = 1.2 or 1.5 *U*_eq_(carrier).

### Crystal data

**Table:**

[Zn(C_14_H_13_N_4_O_2_)_2_]·H_2_O	*F*_000_ = 1288
*M**_r_* = 621.95	*D*_x_ = 1.444 Mg m^−^^3^
Monoclinic, *P*2_1_/*n*	Mo *K*α radiation λ = 0.71073 Å
Hall symbol: -P 2yn	Cell parameters from 2538 reflections
*a* = 9.3794 (10) Å	θ = 2.4–22.4º
*b* = 22.814 (2) Å	µ = 0.91 mm^−^^1^
*c* = 13.9407 (14) Å	*T* = 298 (2) K
β = 106.402 (2)º	Tabular, colourless
*V* = 2861.6 (5) Å^3^	0.32 × 0.20 × 0.16 mm
*Z* = 4	

### Data collection

**Table:**

Bruker SMART CCD area-detector diffractometer	5043 independent reflections
Radiation source: fine-focus sealed tube	2831 reflections with *I* > 2σ(*I*)
Monochromator: graphite	*R*_int_ = 0.077
*T* = 298(2) K	θ_max_ = 25.0º
φ and ω scans	θ_min_ = 1.8º
Absorption correction: multi-scan(SADABS; Bruker, 2000)	*h* = −9→11
*T*_min_ = 0.759, *T*_max_ = 0.868	*k* = −27→23
14227 measured reflections	*l* = −16→16

### Refinement

**Table:**

Refinement on *F*^2^	Secondary atom site location: difference Fourier map
Least-squares matrix: full	Hydrogen site location: inferred from neighbouring sites
*R*[*F*^2^ > 2σ(*F*^2^)] = 0.047	H-atom parameters constrained
*wR*(*F*^2^) = 0.128	*w* = 1/[σ^2^(*F*_o_^2^) + (0.0366*P*)^2^ + 2.1233*P*] where *P* = (*F*_o_^2^ + 2*F*_c_^2^)/3
*S* = 1.02	(Δ/σ)_max_ < 0.001
5043 reflections	Δρ_max_ = 0.38 e Å^−^^3^
379 parameters	Δρ_min_ = −0.35 e Å^−^^3^
Primary atom site location: structure-invariant direct methods	Extinction correction: none

### Special details

**Table:**

Geometry. All e.s.d.'s (except the e.s.d. in the dihedral angle between two l.s. planes) are estimated using the full covariance matrix. The cell e.s.d.'s are taken into account individually in the estimation of e.s.d.'s in distances, angles and torsion angles; correlations between e.s.d.'s in cell parameters are only used when they are defined by crystal symmetry. An approximate (isotropic) treatment of cell e.s.d.'s is used for estimating e.s.d.'s involving l.s. planes.
Refinement. Refinement of *F*^2^ against ALL reflections. The weighted *R*-factor *wR* and goodness of fit *S* are based on *F*^2^, conventional *R*-factors *R* are based on *F*, with *F* set to zero for negative *F*^2^. The threshold expression of *F*^2^ > σ(*F*^2^) is used only for calculating *R*-factors(gt) *etc*. and is not relevant to the choice of reflections for refinement. *R*-factors based on *F*^2^ are statistically about twice as large as those based on *F*, and *R*-factors based on ALL data will be even larger.

### Fractional atomic coordinates and isotropic or equivalent isotropic displacement parameters (Å^2^)

**Table:**

	*x*	*y*	*z*	*U*_iso_*/*U*_eq_	
Zn1	0.09167 (6)	0.14093 (2)	0.53708 (4)	0.04641 (19)	
N1	−0.0550 (4)	0.24903 (15)	0.5747 (2)	0.0414 (9)	
N2	0.0653 (4)	0.23093 (15)	0.5435 (2)	0.0402 (9)	
N3	0.2582 (4)	0.18422 (16)	0.4675 (3)	0.0443 (9)	
N4	0.4647 (4)	0.2484 (2)	0.4049 (3)	0.0592 (11)	
N5	0.2455 (4)	0.02711 (15)	0.5545 (3)	0.0461 (9)	
N6	0.1370 (4)	0.05900 (16)	0.4881 (3)	0.0441 (9)	
N7	−0.0643 (4)	0.12679 (16)	0.3845 (3)	0.0453 (9)	
N8	−0.2259 (5)	0.1065 (2)	0.1898 (3)	0.0636 (12)	
O1	−0.0809 (3)	0.14991 (13)	0.5998 (2)	0.0559 (9)	
O2	−0.2266 (4)	0.32101 (14)	0.6299 (3)	0.0695 (10)	
H2	−0.1514	0.3110	0.6151	0.104*	
O3	0.2353 (3)	0.10277 (13)	0.6631 (2)	0.0512 (8)	
O4	0.4380 (4)	−0.05320 (16)	0.6118 (3)	0.0852 (12)	
H4	0.3692	−0.0353	0.5737	0.128*	
O5	0.7000 (4)	0.3022 (2)	0.3185 (3)	0.1108 (16)	
H5A	0.6191	0.2940	0.3316	0.133*	
H5B	0.6838	0.3291	0.2746	0.133*	
C1	−0.1183 (5)	0.2031 (2)	0.6046 (3)	0.0416 (11)	
C2	−0.2467 (4)	0.21621 (19)	0.6431 (3)	0.0375 (10)	
C3	−0.2931 (5)	0.2732 (2)	0.6550 (3)	0.0465 (11)	
C4	−0.4143 (5)	0.2828 (2)	0.6926 (3)	0.0581 (13)	
H4A	−0.4439	0.3208	0.7018	0.070*	
C5	−0.4885 (5)	0.2362 (3)	0.7158 (3)	0.0608 (14)	
H5	−0.5696	0.2429	0.7403	0.073*	
C6	−0.4470 (5)	0.1797 (2)	0.7039 (4)	0.0610 (14)	
H6	−0.4995	0.1483	0.7194	0.073*	
C7	−0.3265 (5)	0.1706 (2)	0.6685 (3)	0.0511 (12)	
H7	−0.2972	0.1323	0.6612	0.061*	
C8	0.0648 (5)	0.32827 (19)	0.4706 (3)	0.0518 (12)	
H8A	−0.0418	0.3268	0.4508	0.078*	
H8B	0.0985	0.3432	0.4164	0.078*	
H8C	0.0987	0.3535	0.5276	0.078*	
C9	0.1257 (4)	0.26797 (19)	0.4972 (3)	0.0390 (10)	
C10	0.2528 (4)	0.24350 (19)	0.4667 (3)	0.0401 (10)	
C11	0.3614 (5)	0.2760 (2)	0.4387 (3)	0.0472 (11)	
C12	0.3827 (5)	0.3412 (2)	0.4497 (4)	0.0720 (16)	
H12A	0.4853	0.3506	0.4583	0.108*	
H12B	0.3539	0.3543	0.5070	0.108*	
H12C	0.3224	0.3603	0.3908	0.108*	
C13	0.4634 (5)	0.1902 (3)	0.4027 (4)	0.0618 (14)	
H13	0.5317	0.1706	0.3773	0.074*	
C14	0.3641 (5)	0.1584 (2)	0.4370 (3)	0.0526 (12)	
H14	0.3711	0.1177	0.4389	0.063*	
C15	0.2860 (5)	0.0543 (2)	0.6433 (3)	0.0446 (11)	
C16	0.4033 (5)	0.02364 (19)	0.7213 (3)	0.0447 (11)	
C17	0.4738 (5)	−0.0274 (2)	0.7028 (4)	0.0594 (13)	
C18	0.5834 (6)	−0.0531 (2)	0.7792 (4)	0.0769 (17)	
H18	0.6309	−0.0867	0.7664	0.092*	
C19	0.6226 (6)	−0.0297 (3)	0.8728 (5)	0.0829 (18)	
H19	0.6953	−0.0479	0.9235	0.099*	
C20	0.5561 (6)	0.0201 (3)	0.8929 (4)	0.0797 (17)	
H20	0.5847	0.0364	0.9566	0.096*	
C21	0.4463 (5)	0.0461 (2)	0.8181 (4)	0.0611 (14)	
H21	0.3996	0.0795	0.8325	0.073*	
C22	0.1358 (6)	−0.0146 (2)	0.3624 (4)	0.0896 (19)	
H22A	0.2133	−0.0316	0.4154	0.134*	
H22B	0.1733	−0.0061	0.3066	0.134*	
H22C	0.0545	−0.0417	0.3421	0.134*	
C23	0.0833 (5)	0.04049 (19)	0.3982 (3)	0.0436 (11)	
C24	−0.0357 (5)	0.0788 (2)	0.3363 (3)	0.0440 (11)	
C25	−0.1161 (5)	0.0696 (2)	0.2352 (4)	0.0555 (13)	
C26	−0.0901 (6)	0.0220 (3)	0.1688 (4)	0.094 (2)	
H26A	−0.1601	0.0257	0.1039	0.141*	
H26B	−0.1026	−0.0154	0.1969	0.141*	
H26C	0.0091	0.0251	0.1628	0.141*	
C27	−0.2551 (5)	0.1511 (2)	0.2407 (4)	0.0602 (14)	
H27	−0.3334	0.1760	0.2103	0.072*	
C28	−0.1727 (5)	0.1623 (2)	0.3385 (4)	0.0517 (12)	
H28	−0.1940	0.1951	0.3716	0.062*	

### Atomic displacement parameters (Å^2^)

**Table:**

	*U*^11^	*U*^22^	*U*^33^	*U*^12^	*U*^13^	*U*^23^
Zn1	0.0521 (3)	0.0324 (3)	0.0577 (3)	0.0072 (3)	0.0202 (3)	0.0011 (3)
N1	0.040 (2)	0.031 (2)	0.057 (2)	0.0013 (17)	0.0200 (18)	0.0008 (18)
N2	0.039 (2)	0.034 (2)	0.049 (2)	0.0006 (17)	0.0148 (17)	0.0014 (17)
N3	0.042 (2)	0.043 (3)	0.050 (2)	0.0068 (18)	0.0173 (18)	0.0016 (18)
N4	0.046 (2)	0.066 (3)	0.068 (3)	−0.007 (2)	0.020 (2)	0.000 (2)
N5	0.050 (2)	0.031 (2)	0.053 (2)	0.0095 (18)	0.0082 (19)	0.0026 (19)
N6	0.044 (2)	0.039 (2)	0.049 (2)	0.0045 (18)	0.0115 (18)	0.0056 (19)
N7	0.044 (2)	0.036 (2)	0.057 (2)	0.0001 (18)	0.0151 (19)	0.0035 (19)
N8	0.062 (3)	0.064 (3)	0.059 (3)	0.005 (2)	0.007 (2)	0.007 (2)
O1	0.070 (2)	0.0278 (19)	0.083 (2)	0.0063 (16)	0.0433 (18)	0.0034 (16)
O2	0.073 (2)	0.040 (2)	0.108 (3)	0.0033 (18)	0.047 (2)	−0.010 (2)
O3	0.060 (2)	0.038 (2)	0.055 (2)	0.0091 (16)	0.0152 (16)	−0.0037 (16)
O4	0.105 (3)	0.067 (3)	0.070 (2)	0.047 (2)	0.004 (2)	−0.004 (2)
O5	0.083 (3)	0.131 (4)	0.118 (3)	0.007 (3)	0.028 (3)	0.042 (3)
C1	0.047 (3)	0.033 (3)	0.046 (3)	0.002 (2)	0.015 (2)	0.000 (2)
C2	0.038 (2)	0.038 (3)	0.037 (2)	−0.001 (2)	0.0099 (19)	−0.002 (2)
C3	0.048 (3)	0.040 (3)	0.054 (3)	0.003 (2)	0.017 (2)	−0.006 (2)
C4	0.056 (3)	0.051 (3)	0.072 (3)	0.018 (3)	0.025 (3)	−0.005 (3)
C5	0.053 (3)	0.076 (4)	0.060 (3)	0.005 (3)	0.028 (3)	−0.008 (3)
C6	0.061 (3)	0.059 (4)	0.073 (4)	−0.010 (3)	0.036 (3)	−0.008 (3)
C7	0.062 (3)	0.039 (3)	0.057 (3)	0.001 (2)	0.025 (3)	−0.005 (2)
C8	0.062 (3)	0.036 (3)	0.058 (3)	0.006 (2)	0.019 (2)	0.013 (2)
C9	0.040 (3)	0.036 (3)	0.040 (2)	−0.003 (2)	0.009 (2)	0.002 (2)
C10	0.042 (3)	0.036 (3)	0.040 (3)	0.000 (2)	0.008 (2)	0.004 (2)
C11	0.046 (3)	0.048 (3)	0.047 (3)	−0.004 (2)	0.011 (2)	0.000 (2)
C12	0.069 (4)	0.059 (4)	0.091 (4)	−0.020 (3)	0.028 (3)	−0.006 (3)
C13	0.048 (3)	0.069 (4)	0.075 (4)	0.005 (3)	0.027 (3)	−0.008 (3)
C14	0.051 (3)	0.048 (3)	0.062 (3)	0.008 (2)	0.021 (2)	−0.002 (2)
C15	0.043 (3)	0.035 (3)	0.057 (3)	−0.002 (2)	0.016 (2)	0.005 (2)
C16	0.051 (3)	0.031 (3)	0.050 (3)	0.000 (2)	0.010 (2)	0.007 (2)
C17	0.066 (3)	0.043 (3)	0.067 (3)	0.010 (3)	0.017 (3)	0.010 (3)
C18	0.080 (4)	0.055 (4)	0.085 (4)	0.028 (3)	0.007 (3)	0.018 (3)
C19	0.091 (4)	0.062 (4)	0.075 (4)	0.007 (3)	−0.012 (3)	0.022 (3)
C20	0.106 (5)	0.052 (4)	0.065 (4)	0.007 (3)	−0.001 (3)	0.011 (3)
C21	0.074 (4)	0.039 (3)	0.063 (3)	0.001 (3)	0.009 (3)	0.002 (3)
C22	0.122 (5)	0.052 (4)	0.075 (4)	0.032 (3)	−0.006 (3)	−0.015 (3)
C23	0.050 (3)	0.032 (3)	0.051 (3)	−0.003 (2)	0.016 (2)	0.001 (2)
C24	0.043 (3)	0.041 (3)	0.050 (3)	−0.003 (2)	0.016 (2)	0.005 (2)
C25	0.064 (3)	0.047 (3)	0.053 (3)	−0.008 (3)	0.013 (3)	0.002 (3)
C26	0.113 (5)	0.091 (5)	0.056 (3)	0.023 (4)	−0.010 (3)	−0.020 (3)
C27	0.042 (3)	0.065 (4)	0.071 (4)	0.003 (3)	0.012 (3)	0.020 (3)
C28	0.047 (3)	0.046 (3)	0.062 (3)	0.009 (2)	0.016 (3)	0.005 (2)

### Geometric parameters (Å, °)

**Table:**

Zn1—O1	2.055 (3)	C7—H7	0.9300
Zn1—N2	2.073 (3)	C8—C9	1.496 (6)
Zn1—N6	2.075 (4)	C8—H8A	0.9600
Zn1—O3	2.079 (3)	C8—H8B	0.9600
Zn1—N7	2.239 (4)	C8—H8C	0.9600
Zn1—N3	2.283 (3)	C9—C10	1.483 (6)
N1—C1	1.329 (5)	C10—C11	1.401 (6)
N1—N2	1.381 (4)	C11—C12	1.503 (6)
N2—C9	1.288 (5)	C12—H12A	0.9600
N3—C14	1.324 (5)	C12—H12B	0.9600
N3—C10	1.353 (5)	C12—H12C	0.9600
N4—C13	1.329 (6)	C13—C14	1.370 (6)
N4—C11	1.346 (5)	C13—H13	0.9300
N5—C15	1.341 (5)	C14—H14	0.9300
N5—N6	1.374 (4)	C15—C16	1.486 (6)
N6—C23	1.283 (5)	C16—C21	1.391 (6)
N7—C28	1.317 (5)	C16—C17	1.398 (6)
N7—C24	1.350 (5)	C17—C18	1.385 (6)
N8—C27	1.314 (6)	C18—C19	1.360 (7)
N8—C25	1.341 (6)	C18—H18	0.9300
O1—C1	1.270 (5)	C19—C20	1.363 (8)
O2—C3	1.351 (5)	C19—H19	0.9300
O2—H2	0.8200	C20—C21	1.376 (6)
O3—C15	1.264 (5)	C20—H20	0.9300
O4—C17	1.351 (6)	C21—H21	0.9300
O4—H4	0.8200	C22—C23	1.487 (6)
O5—H5A	0.8500	C22—H22A	0.9600
O5—H5B	0.8500	C22—H22B	0.9600
C1—C2	1.482 (6)	C22—H22C	0.9600
C2—C7	1.384 (6)	C23—C24	1.487 (6)
C2—C3	1.395 (6)	C24—C25	1.413 (6)
C3—C4	1.398 (6)	C25—C26	1.492 (7)
C4—C5	1.358 (7)	C26—H26A	0.9600
C4—H4A	0.9300	C26—H26B	0.9600
C5—C6	1.370 (7)	C26—H26C	0.9600
C5—H5	0.9300	C27—C28	1.388 (6)
C6—C7	1.372 (6)	C27—H27	0.9300
C6—H6	0.9300	C28—H28	0.9300
			
O1—Zn1—N2	76.41 (12)	N3—C10—C11	120.2 (4)
O1—Zn1—N6	119.26 (13)	N3—C10—C9	113.9 (4)
N2—Zn1—N6	160.85 (13)	C11—C10—C9	125.9 (4)
O1—Zn1—O3	94.73 (12)	N4—C11—C10	120.1 (4)
N2—Zn1—O3	115.77 (12)	N4—C11—C12	114.2 (4)
N6—Zn1—O3	75.91 (13)	C10—C11—C12	125.6 (4)
O1—Zn1—N7	92.09 (13)	C11—C12—H12A	109.5
N2—Zn1—N7	97.47 (13)	C11—C12—H12B	109.5
N6—Zn1—N7	72.39 (14)	H12A—C12—H12B	109.5
O3—Zn1—N7	146.75 (13)	C11—C12—H12C	109.5
O1—Zn1—N3	148.55 (13)	H12A—C12—H12C	109.5
N2—Zn1—N3	72.21 (13)	H12B—C12—H12C	109.5
N6—Zn1—N3	91.23 (13)	N4—C13—C14	121.6 (5)
O3—Zn1—N3	100.51 (12)	N4—C13—H13	119.2
N7—Zn1—N3	90.03 (12)	C14—C13—H13	119.2
C1—N1—N2	109.9 (3)	N3—C14—C13	121.4 (5)
C9—N2—N1	118.4 (3)	N3—C14—H14	119.3
C9—N2—Zn1	123.6 (3)	C13—C14—H14	119.3
N1—N2—Zn1	115.3 (2)	O3—C15—N5	125.5 (4)
C14—N3—C10	118.2 (4)	O3—C15—C16	119.9 (4)
C14—N3—Zn1	127.5 (3)	N5—C15—C16	114.6 (4)
C10—N3—Zn1	114.0 (3)	C21—C16—C17	117.6 (4)
C13—N4—C11	118.2 (4)	C21—C16—C15	119.2 (4)
C15—N5—N6	109.9 (4)	C17—C16—C15	123.2 (4)
C23—N6—N5	119.7 (4)	O4—C17—C18	118.1 (5)
C23—N6—Zn1	123.9 (3)	O4—C17—C16	122.1 (4)
N5—N6—Zn1	115.9 (3)	C18—C17—C16	119.8 (5)
C28—N7—C24	119.5 (4)	C19—C18—C17	120.8 (5)
C28—N7—Zn1	125.5 (3)	C19—C18—H18	119.6
C24—N7—Zn1	114.9 (3)	C17—C18—H18	119.6
C27—N8—C25	118.7 (4)	C18—C19—C20	120.6 (5)
C1—O1—Zn1	112.3 (3)	C18—C19—H19	119.7
C3—O2—H2	109.5	C20—C19—H19	119.7
C15—O3—Zn1	112.1 (3)	C19—C20—C21	119.4 (5)
C17—O4—H4	109.5	C19—C20—H20	120.3
H5A—O5—H5B	108.9	C21—C20—H20	120.3
O1—C1—N1	125.7 (4)	C20—C21—C16	121.8 (5)
O1—C1—C2	118.5 (4)	C20—C21—H21	119.1
N1—C1—C2	115.8 (4)	C16—C21—H21	119.1
C7—C2—C3	117.5 (4)	C23—C22—H22A	109.5
C7—C2—C1	119.6 (4)	C23—C22—H22B	109.5
C3—C2—C1	122.9 (4)	H22A—C22—H22B	109.5
O2—C3—C2	122.6 (4)	C23—C22—H22C	109.5
O2—C3—C4	117.0 (4)	H22A—C22—H22C	109.5
C2—C3—C4	120.3 (4)	H22B—C22—H22C	109.5
C5—C4—C3	119.4 (5)	N6—C23—C22	122.0 (4)
C5—C4—H4A	120.3	N6—C23—C24	113.4 (4)
C3—C4—H4A	120.3	C22—C23—C24	124.6 (4)
C4—C5—C6	121.7 (4)	N7—C24—C25	119.4 (4)
C4—C5—H5	119.1	N7—C24—C23	114.2 (4)
C6—C5—H5	119.1	C25—C24—C23	126.4 (4)
C5—C6—C7	118.6 (5)	N8—C25—C24	120.0 (5)
C5—C6—H6	120.7	N8—C25—C26	113.7 (4)
C7—C6—H6	120.7	C24—C25—C26	126.3 (5)
C6—C7—C2	122.5 (5)	C25—C26—H26A	109.5
C6—C7—H7	118.8	C25—C26—H26B	109.5
C2—C7—H7	118.8	H26A—C26—H26B	109.5
C9—C8—H8A	109.5	C25—C26—H26C	109.5
C9—C8—H8B	109.5	H26A—C26—H26C	109.5
H8A—C8—H8B	109.5	H26B—C26—H26C	109.5
C9—C8—H8C	109.5	N8—C27—C28	122.0 (5)
H8A—C8—H8C	109.5	N8—C27—H27	119.0
H8B—C8—H8C	109.5	C28—C27—H27	119.0
N2—C9—C10	113.6 (4)	N7—C28—C27	120.3 (5)
N2—C9—C8	122.5 (4)	N7—C28—H28	119.9
C10—C9—C8	123.8 (4)	C27—C28—H28	119.9
			
C1—N1—N2—C9	169.0 (4)	C3—C4—C5—C6	0.5 (7)
C1—N1—N2—Zn1	7.0 (4)	C4—C5—C6—C7	0.6 (8)
O1—Zn1—N2—C9	−166.9 (3)	C5—C6—C7—C2	−0.9 (7)
N6—Zn1—N2—C9	−20.1 (6)	C3—C2—C7—C6	0.2 (6)
O3—Zn1—N2—C9	104.4 (3)	C1—C2—C7—C6	−179.5 (4)
N7—Zn1—N2—C9	−76.6 (3)	N1—N2—C9—C10	−179.5 (3)
N3—Zn1—N2—C9	11.0 (3)	Zn1—N2—C9—C10	−19.0 (5)
O1—Zn1—N2—N1	−5.9 (2)	N1—N2—C9—C8	−3.9 (6)
N6—Zn1—N2—N1	140.9 (4)	Zn1—N2—C9—C8	156.6 (3)
O3—Zn1—N2—N1	−94.6 (3)	C14—N3—C10—C11	−2.9 (6)
N7—Zn1—N2—N1	84.5 (3)	Zn1—N3—C10—C11	171.5 (3)
N3—Zn1—N2—N1	172.1 (3)	C14—N3—C10—C9	177.0 (3)
O1—Zn1—N3—C14	177.5 (3)	Zn1—N3—C10—C9	−8.6 (4)
N2—Zn1—N3—C14	173.7 (4)	N2—C9—C10—N3	17.1 (5)
N6—Zn1—N3—C14	−16.1 (4)	C8—C9—C10—N3	−158.5 (4)
O3—Zn1—N3—C14	59.8 (4)	N2—C9—C10—C11	−162.9 (4)
N7—Zn1—N3—C14	−88.5 (4)	C8—C9—C10—C11	21.5 (6)
O1—Zn1—N3—C10	3.7 (4)	C13—N4—C11—C10	−2.5 (6)
N2—Zn1—N3—C10	−0.1 (3)	C13—N4—C11—C12	173.2 (4)
N6—Zn1—N3—C10	170.2 (3)	N3—C10—C11—N4	5.2 (6)
O3—Zn1—N3—C10	−114.0 (3)	C9—C10—C11—N4	−174.7 (4)
N7—Zn1—N3—C10	97.8 (3)	N3—C10—C11—C12	−169.9 (4)
C15—N5—N6—C23	179.7 (4)	C9—C10—C11—C12	10.1 (7)
C15—N5—N6—Zn1	7.7 (4)	C11—N4—C13—C14	−2.4 (7)
O1—Zn1—N6—C23	92.9 (3)	C10—N3—C14—C13	−1.9 (6)
N2—Zn1—N6—C23	−49.5 (6)	Zn1—N3—C14—C13	−175.4 (3)
O3—Zn1—N6—C23	−179.6 (4)	N4—C13—C14—N3	4.7 (7)
N7—Zn1—N6—C23	10.6 (3)	Zn1—O3—C15—N5	−5.1 (5)
N3—Zn1—N6—C23	−79.1 (3)	Zn1—O3—C15—C16	173.2 (3)
O1—Zn1—N6—N5	−95.5 (3)	N6—N5—C15—O3	−1.6 (6)
N2—Zn1—N6—N5	122.1 (4)	N6—N5—C15—C16	−180.0 (3)
O3—Zn1—N6—N5	−8.0 (3)	O3—C15—C16—C21	5.8 (6)
N7—Zn1—N6—N5	−177.8 (3)	N5—C15—C16—C21	−175.7 (4)
N3—Zn1—N6—N5	92.6 (3)	O3—C15—C16—C17	−174.3 (4)
O1—Zn1—N7—C28	54.9 (4)	N5—C15—C16—C17	4.1 (6)
N2—Zn1—N7—C28	−21.7 (4)	C21—C16—C17—O4	178.7 (4)
N6—Zn1—N7—C28	175.0 (4)	C15—C16—C17—O4	−1.1 (7)
O3—Zn1—N7—C28	156.8 (3)	C21—C16—C17—C18	−0.9 (7)
N3—Zn1—N7—C28	−93.7 (4)	C15—C16—C17—C18	179.3 (5)
O1—Zn1—N7—C24	−128.8 (3)	O4—C17—C18—C19	−178.9 (5)
N2—Zn1—N7—C24	154.6 (3)	C16—C17—C18—C19	0.7 (8)
N6—Zn1—N7—C24	−8.7 (3)	C17—C18—C19—C20	−1.0 (9)
O3—Zn1—N7—C24	−26.9 (4)	C18—C19—C20—C21	1.4 (9)
N3—Zn1—N7—C24	82.6 (3)	C19—C20—C21—C16	−1.6 (8)
N2—Zn1—O1—C1	3.6 (3)	C17—C16—C21—C20	1.3 (7)
N6—Zn1—O1—C1	−164.5 (3)	C15—C16—C21—C20	−178.9 (5)
O3—Zn1—O1—C1	119.0 (3)	N5—N6—C23—C22	−0.6 (6)
N7—Zn1—O1—C1	−93.6 (3)	Zn1—N6—C23—C22	170.7 (4)
N3—Zn1—O1—C1	−0.1 (4)	N5—N6—C23—C24	178.5 (3)
O1—Zn1—O3—C15	125.7 (3)	Zn1—N6—C23—C24	−10.2 (5)
N2—Zn1—O3—C15	−157.1 (3)	C28—N7—C24—C25	4.0 (6)
N6—Zn1—O3—C15	6.7 (3)	Zn1—N7—C24—C25	−172.5 (3)
N7—Zn1—O3—C15	24.6 (4)	C28—N7—C24—C23	−176.6 (4)
N3—Zn1—O3—C15	−81.9 (3)	Zn1—N7—C24—C23	6.8 (4)
Zn1—O1—C1—N1	−0.9 (5)	N6—C23—C24—N7	1.2 (5)
Zn1—O1—C1—C2	176.8 (3)	C22—C23—C24—N7	−179.7 (4)
N2—N1—C1—O1	−4.1 (6)	N6—C23—C24—C25	−179.4 (4)
N2—N1—C1—C2	178.2 (3)	C22—C23—C24—C25	−0.4 (7)
O1—C1—C2—C7	−2.9 (6)	C27—N8—C25—C24	0.8 (7)
N1—C1—C2—C7	175.0 (4)	C27—N8—C25—C26	−177.9 (5)
O1—C1—C2—C3	177.4 (4)	N7—C24—C25—N8	−3.9 (7)
N1—C1—C2—C3	−4.7 (6)	C23—C24—C25—N8	176.8 (4)
C7—C2—C3—O2	−178.0 (4)	N7—C24—C25—C26	174.6 (5)
C1—C2—C3—O2	1.7 (7)	C23—C24—C25—C26	−4.7 (8)
C7—C2—C3—C4	0.9 (6)	C25—N8—C27—C28	2.3 (7)
C1—C2—C3—C4	−179.4 (4)	C24—N7—C28—C27	−1.1 (6)
O2—C3—C4—C5	177.7 (4)	Zn1—N7—C28—C27	175.1 (3)
C2—C3—C4—C5	−1.3 (7)	N8—C27—C28—N7	−2.2 (7)
